# Special Issue: Genetics of Biodegradation and Bioremediation

**DOI:** 10.3390/genes11040441

**Published:** 2020-04-17

**Authors:** Eduardo Santero, Eduardo Díaz

**Affiliations:** 1 Department of Molecular Biology and Biochemical Engineering. Centro Andaluz de Biología del Desarrollo, Universidad Pablo de Olavide/Consejo Superior de Investigaciones Científicas/Junta de Andalucía, 41013 Seville, Spain; 2 Department of Microbial and Plant Biotechnology, Centro de Investigaciones Biológicas Margarita Salas, Agencia Estatal Consejo Superior de Investigaciones Científicas, 28040 Madrid, Spain; ediaz@cib.csic.es

**Keywords:** biodegradation, bioremediation, valorisation, catabolic pathway, mobile DNA, anaerobic biodegradation, gene regulation

## Abstract

Many different biodegradation pathways, both aerobic and anaerobic, have already been characterised, and the phylogenetic relationships among catabolic genes within the different types of pathways have been studied. However, new biodegradation activities and their coding genes are continuously being reported, including those involved in the catabolism of emerging contaminants or those generally regarded as non-biodegradable. Gene regulation is also an important issue for the efficient biodegradation of contaminants. Specific induction by the substrate and over-imposed global regulatory networks adjust the expression of the biodegradation genes to the bacterial physiological needs. New biodegradation pathways can be assembled in a particular strain or in a bacterial consortium by recruiting biodegradation genes from different origins through horizontal gene transfer. The abundance and diversity of biodegradation genes, analysed by either genomic or metagenomic approaches, constitute valuable indicators of the biodegradation potential of a particular environmental niche. This knowledge paves the way to systems metabolic engineering approaches to valorise biowaste for the production of value-added products.

Industrial activity has resulted in the continuous production of huge amounts of contaminant molecules, many of which are very persistent in nature because of their stable chemical structure, low biodegradability, or toxicity, thus leading to their accumulation in the environment to concentrations that may affect natural populations of living beings. However, some microorganisms, mainly bacteria, have evolved to metabolise these contaminants, in many cases using them as nutrient and/or energy sources. Besides the importance of these biodegrading strains to mitigate contamination of natural areas, the study of their biodegradation pathways has revealed very interesting features of the catabolic activities involved, how they evolve in really short periods of time, and how they can be spread among different bacteria. 

This book reprints 12 contributions that show recent advances in our knowledge of different aspects of biodegradation of particular contaminants in different bacteria at biochemical, genetic, and genomic levels. 

One of the most abundant contaminants on Earth is the xenobiotic plastic polyethylene terephthalate (PET). It is chemically very stable and highly refractory to microbial biodegradation. However, a number of bacteria have been recently reported that have PET hydrolase activities and further ability to use the resulting monomers (ethylene glycol and terephthalate) as carbon and energy sources. These biodegradation activities that allow PET to be used as a feedstock and the possibility of biosynthesising PET from sustainable substrates in a circular bio-PET economy not dependent on fossil fuels are reviewed by Salvador et al. [1].

Two additional reviews deal with related aromatic contaminants that can be found in crude oils. The review by Phale et al. [2] focuses on the mechanisms of assembly of biodegradation pathways of the well-known biaromatic naphthalene and its derivative, carbaryl, that have apparently evolved by recruiting different catabolic activities via horizontal gene transfer through mobile genetic elements. Tetralin, found in oil but also industrially produced from naphthalene or anthracene, is similar to naphthalene, but one of the rings is alicyclic. The biodegradation pathway by which a conventional enzymatic team involved in the biodegradation of one aromatic ring is able to metabolise both the aromatic and the alicyclic ring of tetralin is thoroughly reviewed at biochemical, genetics, genomics, and gene regulation levels [3].

Steroids are complex molecules composed of four alicyclic rings, which are produced by eukaryotic cells and also synthesised by the pharma industry. Although they are very recalcitrant due to their hydrophobic nature and the absence of functional groups, several steroid-metabolising bacteria have been isolated, and their biodegradation pathways characterised. The different strategies to metabolise steroids by different bacteria, including a comparative analysis of the genes responsible for these activities, are reviewed by Olivera and Luengo [4]. Steroid biodegradation is also the focus of an original research paper by Ibero et al. [5], where the testosterone biodegradation pathway of a *Novosphingobium* strain is inferred from in silico genomic analysis and subsequently confirmed experimentally.

Besides bearing the genes that code for biodegradation capability, it is crucial for an efficient catabolic process that these genes are expressed at sufficiently high levels and only when the substrate to be metabolised is present in order to prevent the wasteful production of catabolic enzymes and reactions when they are not needed or advantageous. Regulatory systems, which may evolve independently of the catabolic genes, then have to adjust the range of molecules to which they respond to the range of molecules that can be metabolised by the regulated catabolic pathway in order to prevent gratuitous induction by a molecule that cannot be metabolised. Besides this specific regulation, catabolic pathways may be controlled by an over-imposed regulatory circuit, resulting in a catabolite repression phenomenon that prevents their expression when the bacterium can use other more favourable nutrients. The importance of a regulatory system leading to sufficient expression of the catabolic genes is shown by Igeño et al. [6], who demonstrate that a strain of *Pseudomonas pseudoalcaligenes* with the potential to use furfuryl alcohol, furfural, and furoic acid as carbon and energy sources can only grow on these substrates when it gains a particular mutation in a regulatory gene coding for an AraC-type transcriptional activator. Durante-Rodríguez et al. [7] documented the regulation of an anaerobic benzoate biodegradation pathway by identifying the *bzdR* repressor gene in different *Azoarcus*/*Aromatoleum* strains and characterising the repressor binding and repressor complex formation at the target promoter-regulatory region. The relevance of the specificity of the regulatory protein for the response to a particular inducer and how this specificity may be tuned to create variants that respond synergistically to several effectors is shown by Tumen-Velasquez et al. [8] by comparing the effector specificity of two very similar LysR-type activators, the CatM and BenM paralogs in *Acinetobacter baylyi* ADP1. Finally, the sophisticated regulatory system consisting of three regulatory circuits that control the expression of the tetralin biodegradations genes is reviewed by Floriano et al. [3].

Genes coding for a catabolic pathway are always in a genomic context, either chromosomal or extra-chromosomal (e.g., plasmid, transposon, integrative-conjugative element), and are expressed in bacteria with different metabolic capabilities and physiologies. Exploring the genomic contexts of catabolic genes becomes essential to know the potential for mobility of such genes, as well as to understand the physiology and metabolism of the host strain, including the biodegradation potential of other contaminants, and the similarities and differences among different biodegradation strains. Genomic analysis of a strain able to degrade the highly toxic cyanide has revealed its potential to also degrade furfural [5]. Genomic analysis by Imperato et al. [9] of two naphthalene-degrading *Pseudomonas* strains isolated from an oil-contaminated field revealed that their genomes also bear genes for the degradation of other contaminants such, as alkanes, BTEX (acronym for benzene, toluene, ethyl benzene and xylenes), anthranilate, or terephthalate. Whereas both strains contain a plasmid, only the large plasmid of one of the strains bear the whole set of *nah* genes required to use naphthalene as the carbon and energy source. The other strain apparently has the *nah* genes associated with mobile DNA sequences but integrated into the chromosome. Naphthalene biodegradation genes associated with mobile DNA elements is also reviewed by Phale et al [2]. Genomic analysis of *Sphingopyxis lindanitolerans*, a strain able to degrade the γ-hexachlorocyclohexane pesticide, by Kaminski et al. [10] revealed that it contains two plasmids that bear different *lin* genes associated with insertion sequences, which together encode the entire lindane biodegradation pathway. Genomic analysis of ten strains isolated from biphenyl-contaminated sites by Hirose et al. [11] showed the very high incidence of *bph* genes associated with integrative conjugative elements and revealed the molecular basis of its dissemination potential by lateral transfer among different bacteria. 

Bacteria are not alone in nature, and they interact with each other. In many instances, biodegradation is achieved by a consortium of microorganisms that may have similar or even complementary activities, rather than by a single population. This cooperation is even more evident when the pollution is caused by a mixture of contaminants, such as that produced by spills or leakage of petroleum hydrocarbons. In these cases, bioremediation of a polluted site requires the concerted action of different populations of bacteria with different catabolic capacities. The characterisation of these bacterial consortia is crucial for the understanding of bioremediation processes of complex mixtures of pollutants. The paper by Garrido-Sanz et al. [12] analyses the population composition of a consortium isolated from a diesel-contaminated site, showing how this composition changes when growing in the presence of a specific hydrocarbon, and assigning functions to particular members of the consortium.

In summary, all the contributions presented in this book highlight the importance of unravelling the genetic determinants responsible for the astonishing genomic plasticity of biodegradation strains to rapidly adapt to the presence of new molecules that are continuously released to the environment, some of which are emerging priority pollutants. Moreover, these studies pave the way to systems metabolic engineering approaches to valorise some abundant contaminants as feedstock for the production of value-added products in a circular bioeconomy strategy. 
